# Aerobic exercise and metformin on intermuscular adipose tissue (IMAT): insights from multimodal MRI and histological changes in prediabetic rats

**DOI:** 10.1186/s13098-023-01183-x

**Published:** 2023-10-30

**Authors:** Fuyao Yu, Chuan Xing, Yiping Fan, Yanping Liu, Peng Su, Qiuhua Yang, Yanbin Dong, Yang Hou, Shinong Pan

**Affiliations:** 1 Department of Radiology, Shengjing Hospital of China Medical University, 36 Sanhao Street, Heping District, Shenyang, 110004 China; 2 Department of Endocrinology, The People’s Hospital of Liaoning Province, Shenyang, China; 3 Department of Nuclear Medicine, The Fourth Affiliated Hospital of China Medical University, Shenyang, China; 4 Department of Gastroenterology and Medical Research Center, Shengjing Hospital of China Medical University, Shenyang, China; 5 Vascular Biology Center, Medical College of Georgia, Augusta University, Augusta, GA 30912 USA; 6 Department of Medicine, Georgia Prevention Institute, Medical College of Georgia, Augusta, GA 30912 USA

**Keywords:** Adipose tissue, Metformin, Aerobic exercise, Prediabetes, Multimodal imaging, Magnetic-resonance imaging

## Abstract

**Background:**

Physical exercise is the first-line intervention for prediabetes, and metformin is the most widely used oral insulin-sensitizing agent. Moreover, intermuscular adipose tissue (IMAT) directly affects insulin resistance by helping maintain glucose homeostasis. Here, we evaluated the effects of moderate aerobic exercise and/or metformin on histological IMAT parameters in non-streptozotocin-induced prediabetes.

**Methods:**

Male Wistar rats with prediabetes fed a high-fat diet and high-sugar drinks were randomly assigned to high-fat diet (PRE), metformin (MET), moderate aerobic exercise (EXE), combined therapy (EMC), or EMC + compound-c (EMA) groups for 4 weeks. Multimodal magnetic resonance imaging (MRI) was then performed, and tissue-specific inflammation and energy and lipid metabolism were evaluated in IMAT.

**Results:**

The EXE group had lower inflammatory factor levels, lipid metabolism, and mitochondrial oxidative stress, and shorter IMAT adipocyte diameters than the MET group. The MET group exhibited lower IL-1β and Plin5 expression than the PRE group. Furthermore, the IMAT of the EMC group had lower TNF-α and phosphorylated NF-κB levels and higher GLUT1 and GLUT4 expression than the PRE group. Multimodal MRI revealed significant changes in transverse-relaxation time 2, apparent diffusion coefficient, and fractional anisotropy values in the IMAT and muscles, as well as lower IMAT% values in the EXE and EMC groups than in the MET and PRE groups.

**Conclusion:**

Moderate aerobic exercise training can effectively improve IMAT function and structure via the AMP-activated protein kinase pathway in prediabetes. Combining metformin with moderate aerobic exercise might elicit modest synergy, and metformin does not counterbalance the beneficial effects of exercise.

**Supplementary Information:**

The online version contains supplementary material available at 10.1186/s13098-023-01183-x.

## Introduction

Prediabetes involves abnormally high blood glucose levels below the diagnostic threshold for type-2 diabetes mellitus (T2DM) [1]. Approximately 5–10% of prediabetic patients develop T2DM each year [2]. Muscles and adipose tissue are insulin-sensitive organs that help maintain glucose homeostasis. Intermuscular adipose tissue (IMAT) is characterized as the adipose tissue that is intricately interspersed among and surrounding groups of skeletal muscles. The proportion of IMAT was elevated in individuals with T2DM and metabolic syndrome compared to their healthy counterparts; this escalation was integrally linked to insulin resistance and an age-associated decline in physical functionality [3]. Additionally, IMAT could directly modulate insulin resistance (IR), independently of other body fat depots [4], by secreting inflammatory cytokines and expressing the perilipin 5 (*Plin5*) gene [5, 6]. Nonetheless, the role that IMAT plays in the prediabetic stage and the metabolic changes that occur when it is targeted therapeutically is unclear. Therefore, it's vital to target IMAT to prevent the progression of diabetes from prediabetes.

The American Diabetes Association recommends metformin as a preventative for diabetes [7, 8]. Metformin is a first-line T2DM drug that can activate AMP-activated protein kinase (AMPK) [9, 10] and enhance insulin activity in the liver and skeletal muscles [11]. Meanwhile, aerobic exercise could increase the translocation of the transmembrane proteins FAT/CD36 and GLUT-4 in skeletal muscles by activating AMPK to promote fatty acid and glucose uptake [12, 13]. Thus, combining metformin with aerobic exercise in prediabetes may be more effective than either intervention alone. However, recent data challenge this notion [14], and metformin might antagonize insulin while sensitizing the body to the effects of exercise [15]. Furthermore, combined intervention with metformin and exercise significantly improved the area-under-the-curve (AUC) glucose values and percentage of hyperglycemic peaks in prediabetic populations [16]. Further research indicated that the conflicting results might stem from the different activation mechanisms of AMPK by exercise and metformin [14]. Briefly, aerobic exercise primarily activated AMPK by accelerating energy metabolism and stimulating ATP consumption [17], while metformin activated AMPK by mimicking a low-energy environment within tissues [18]. As such, the impact of combined metformin and exercise therapy on IMAT in prediabetes remains unclear. Taken together, the main purpose of the present study was to test whether exercise and metformin, either alone or in combination, could positively influence IMAT in the prediabetic status, the early stage of the development of diabetes.

Multimodal magnetic-resonance imaging (MRI) can facilitate non-invasive measurements of biological changes. Specifically, Dixon and magnetic resonance spectroscopy (MRS) sequences can measure IMAT and intramyocellular lipids (IMCLs) in skeletal muscles, respectively. Such differentiation is crucial considering the distinct role of IMAT in prediabetic metabolic profiles. At the histological level, transverse-relaxation time 2 (T2)-mapping sequences are utilized to derive T2 values, which are applied to analyze biochemical and metabolic information in target tissues. This approach might emerge as a screening test for the early detection of metabolic diseases, such as diabetes [19]. Furthermore, diffusion-tensor imaging (DTI) is employed to track muscle fibers and to calculate both apparent diffusion coefficient (ADC) and fractional anisotropy (FA) values. ADC values reflect the diffusion ability of water molecules within biological tissues, indicating tissue cell density and structure. FA values, however, represent the directional dependence of this diffusion, providing insights into the microstructure of tissues, including the integrity and direction of muscle fibers [20]. Together, these multimodal MRI techniques composed of various MRI sequences hold promise for elucidating the critical effects of aerobic exercise and metformin on IMAT in prediabetics.

Herein, in this study, we integrated metabolic assessments with multimodal MRI parameters to comprehensively explore the impact of moderate aerobic exercise and/or metformin on IMAT in a non-STZ-induced prediabetic rat model.

## Methods

### Animal model and grouping protocol

Male Wistar rats (200 ± 10 g; Beijing Huafukang Biotechnology, Beijing, China) were housed under a 12:12 h light/dark cycle, at a constant temperature of 24 ± 1 °C, with ad libitum access to food and water, the total of 36 rats were divided: 6 were allocated to the Control Group (CON) and the remaining 30 to the prediabetic group. Rats were assigned to the groups using a simple randomization method. The PRE group was fed a diet supplemented with 60% lard (Beijing Huafukang Biotechnology, Beijing, China). From weeks 6–10, rats in the PRE group were provided 30% sucrose solution instead of water. By the 11th week, the prediabetes induction in the PRE group was confirmed. The body weights and fasting blood glucose (FBG) levels of all rats were measured weekly. Our model is also consistent with a previous model in which rats in the PRE group developed prediabetes [21]. Specifically, pre-diabetic rats exhibited moderately increased adiposity, impaired glucose tolerance, and insulin resistance.

These rats were then further subdivided into five distinct groups of 6 rats each: diabetic control (PRE), moderate-aerobic exercise (EXE), metformin-treatment (MET), aerobic-exercise + metformin-treatment + AMPK pathway-inhibitor (EMA), and-aerobic exercise + metformin-treatment (EMC) groups (*n* = 6 rats/group). The rats were assigned to these subgroups randomly, ensuring an even distribution of characteristics like body weight and FBG levels.

### Exercise protocol and drug administration

In week 11, the EXE, EMA, and EMC groups, comprising a total of 18 rats, initiated a 4-week aerobic-exercise training. The rats were placed on a treadmill for 4 weeks (5 d/week, 60 min/d); the speed was gradually increased to 16 m/min (including a 5 min warm-up period at 10 m/min), equating to moderate aerobic exercise [22]. To eliminate potential influences of handling and confinement, rats that did not engage in physical exercise were placed on a stationary treadmill for an equivalent duration. Before aerobic-exercise training began, the rats were trained for 10 min at 10 m/min with a 5° inclination angle for 3 d.

Metformin (300 mg kg^−1^ day^−1^) was administered intragastrically to the MET, EMA, and EMC groups (*n* = 18) from weeks 11–14 [23]. Rats in the control, PRE, and EXE groups (*n* = 12) were administered the same amount of ultra-pure water. Exercise intervention was performed 2 h after administering daily drug treatments. To the EMA group, compound-c (20 mg/[kg d]) [24] was injected daily into the muscles of both lower limbs after exercise from weeks 11–14. The remaining groups were administered an equivalent volume of 0.9% NaCl solution. Metformin was dissolved in ultra-pure water, and compound-c was dissolved in dimethyl sulfoxide (Merck, Darmstadt, Germany).

### Blood testing and tissue sampling

Fasting blood glucose levels of all rats (*n* = 36) were measured weekly using a blood glucose-monitoring system (Accu-Check; Roche, Basel, Switzerland). After overnight fasting (weeks 10 and 14), all rats were gavaged with 1.5 g/kg anhydrous glucose, and oral glucose tolerance tests (OGTTs) were performed; blood glucose levels were measured after 0, 30, 60, and 120 min post-gavage. During week 10, 300 μL of blood was collected from the medial canthus vein of the right eye of each rat, and plasma was isolated by centrifugation (10,000×*g* for 5 min).

After the last session of exercise and metformin interventions, all rats (*n* = 36) were anesthetized using a mixture of ketamine (100 mg/kg i.p.) and dexmedetomidine (0.5 mg/kg i.p.) until loss of reflexes [25]. Blood (1.5 mL) was obtained from the heart, plasma was isolated via centrifugation (10,000×*g* for 5 min), and plasma-insulin levels were measured via enzyme-linked immunosorbent assays. Fresh rat quadriceps femoris were placed under an anatomical microscope and dissected on ice for 1–1.5 min. IMAT and skeletal muscles were separated, confirmed not to contain other tissues [5]. A portion was stored at − 80 °C, while another portion was fixed with 4% formaldehyde.

### Multimodal MRI

All Rats (*n* = 36) were placed in an 30.T MRI (Philips Health Tech., Amsterdam, Netherlands). Each rat was positioned prone inside the MRI scanner and an elbow joint coil was used for scanning. The scanning sequence included T2 mapping, DTI, magnetic-resonance spectroscopy (MRS), and MR-Dixon analysis (Table 1). Furthermore, we acquired axial T1WI, T2WI, and sagittal T2WI scans to obtain the localization required for DTI and MRS. DTI images were obtained in six diffusion directions, and 600 mm/s^2^ was used as the b value. To draw regions of interest (ROIs), Philips Research Imaging Development Environment (PRIDE) software (v.4.1.V3) and Mimics Research software (v.21.0) were employed for image post-processing.

**Table 1:** Magnetic-resonance sequence-acquisition parameters

Scanning parameter	T1WI	T2WI	DTI	T2 mapping	Dixon	MRS
TR	120	2500	2500	1500	9.1	2000
TE	10	80	53	9.0	1.33	50
FOV (mm)	100 × 120 × 60	100 × 121 × 66	120 × 90 × 60	90 × 121 × 39	100 × 100 × 25	30 × 30 × 30
SNR	1.02	0.96	1.0	1.0	1.0	–
NSA	3	3	2	2	2	128
Scan time (min)	1.46	3.15	5.15	5.45	0.09	4.52
Slices	20	20	20	12	10	–

*DTI* diffusion-tensor imaging, *FOV* field of view, *MRS* magnetic-resonance spectroscopy, *NSA* number of signals averaged, *SNR* signal: noise ratio, *TE* time of echo, *TR* time of repetition

**Fig. 1:**
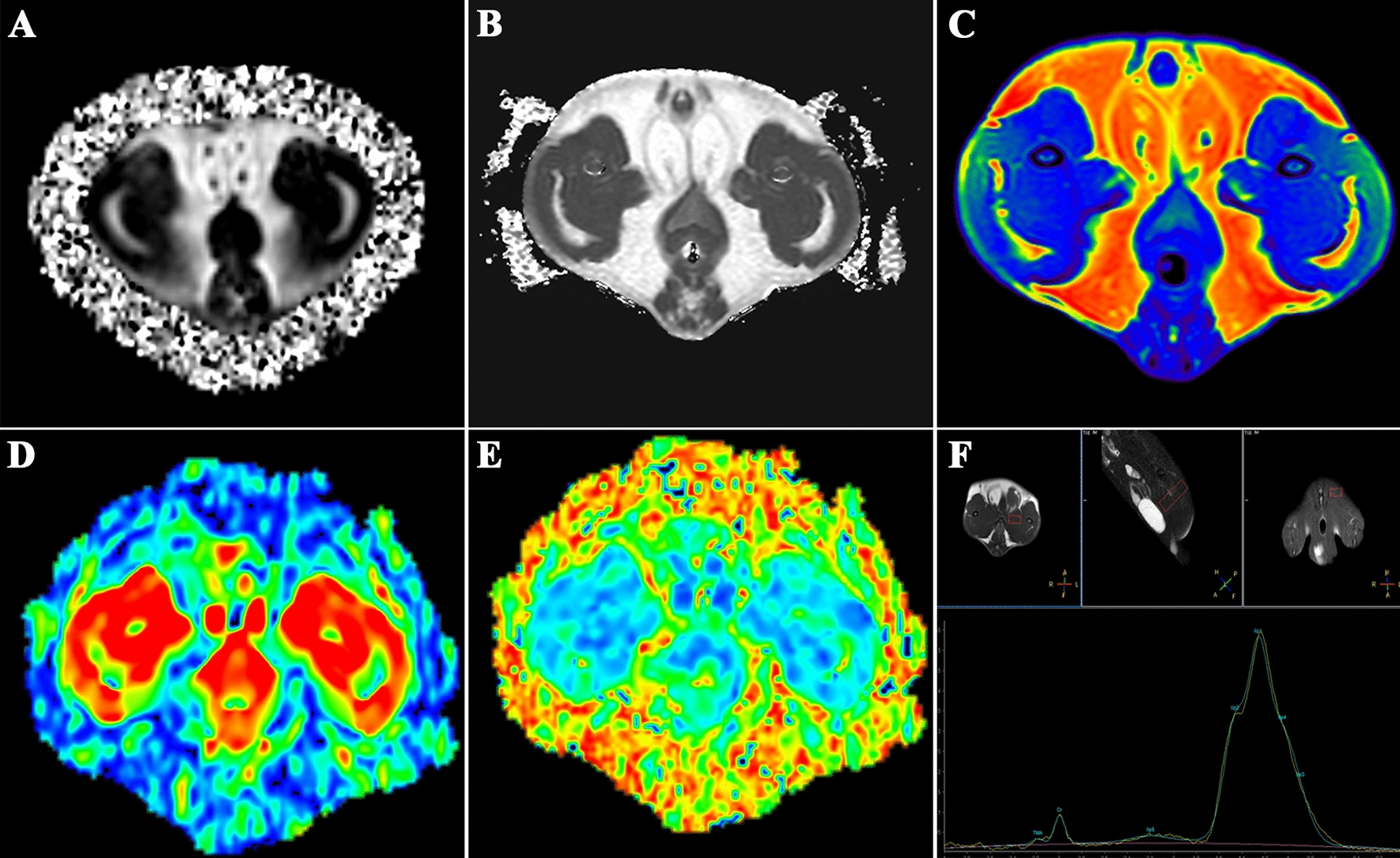
Characterization of multimodal magnetic-resonance imaging in the middle thigh femur of rats with prediabetes. **A** Chemical-shift-encoded water-fat sequence image (Dixon method) in the middle thigh femur. **B** Transverse relaxation time 2 (T2)-mapping image of the middle thigh femur. **C** Artificial-color map of T2-mapping image of the middle thigh femur. **D**, **E** Artificial-color map of a diffusion-tensor imaging image of the middle thigh femur. **F** Magnetic-resonance spectroscopy image of a quadriceps femur

We also measured (1) the percentage of IMAT in skeletal muscles, (2) T2 values in skeletal muscles and IMATs, (3) ADC and FA values in skeletal muscles and IMATs, and (4) intramyocellular lipid/creatinine (IMCL/Cr) ratios in skeletal muscles (Fig. 1).

### Quantitative image analysis

To ensure the reliability and consistency of the multimodal MRI parameters, imaging-data postprocessing and analysis were conducted on a computer workstation (Philips Healthcare, Best, The Netherlands) by three experienced radiologists (Y.F., F.Y, and P.S.) blinded to the study design and the subjects’ clinical information. To assess inter-examiner reproducibility and the reliability of the MRI quantitative parameters, all radiologists independently performed the measurements twice, with at least a 2-week interval between each measurement. ROIs were delineated within muscles and IMATs (avoiding vertebrae, fasciae, subcutaneous fat, and blood vessels). Interclass-correlation coefficients were determined to assess the precision of the multimodal MRI parameters.

### Immunohistochemical analysis

All rats’ (*n* = 36) IMAT samples were fixed with 4% paraformaldehyde and embedded in paraffin. The sections (5 μm) were stained with antibodies against tumor necrosis factor-α (TNF-α), interleukin (IL)-6, and IL-1β. After the tissue sections were dewaxed with xylene and rehydrated with a graded-ethanol series, antigens were repaired in 0.01 mol/L ethylenediaminetetraacetic acid (pH 8.0) for 1.5 min in a high-pressure sterilizer. The sections were then incubated with hydrogen peroxide for 10 min and non-specific staining was reduced using normal goat serum. Subsequently, the sections were incubated with anti-TNF-α (1:200, Affinity Biosciences, AF7014, Jiangsu, China), IL-6 (1:50, Protein-tech, 21865-1-AP, Wuhan, China), or IL-1β (1:500, Affinity Biosciences, AF5103, Jiangsu, China) antibodies overnight at 4 °C, followed by complementary secondary antibodies. We used 3-diaminobenzidine for visualization. The sections were counterstained with hematoxylin, dehydrated using a gradual alcohol gradient, and sealed with neutral gum. Staining was visualized using an inverted light microscope (Nikon, TS100, Tokyo, Japan) and quantified with ImageJ software (v1.48; NIH, Bethesda, MD). For each rat, each antibody staining was repeated on three distinct sections, and the staining intensity of these sections was averaged for further analysis.

### Histological analysis

Three Paraffin-embedded IMAT sections (5 μM) from each rat were stained with hematoxylin and eosin for histopathological examination; the average adipocyte diameter of each rat was quantified by ImageJ software. From each rat, three sections of fresh muscle samples were embedded in optimal cutting temperature compound (OCT) and dissected into 10 μM slices. These sections were stained with oil red O solution (3 mg/mL) and rinsed as described [26]. The average IMAT content from the three sections of each rat’s skeletal muscles was then quantified using ImageJ software [27].

### Immunoblotting

Total protein was extracted from IMATs using the Minute Total Protein Extraction Kit for Adipose Tissues (Invent, AT-022, Minnesota, USA) and quantified using a BCA Detection Assay Kit (Beyotime, P0010S, Shanghai, China). To ensure uniformity in protein loading across all samples, the protein concentration of each sample was carefully adjusted so that the same amount of protein (20 μg) in each sample (200 μg IMAT from quadriceps) was denatured in a loading buffer comprising 2% SDS, 10% glycerol, 1% β-mercaptoethanol, 0.004% Bromophenol blue, and 62.5 mM Tris–HCL (pH 6.8) at 100 °C for 5 min and separated by 10% sodium dodecyl sulfate–polyacrylamide gel electrophoresis. Proteins were transferred to polyvinylidene fluoride membranes, blocked with 5% skim milk for 2 h, and incubated overnight at 4 °C with an antibody against rabbit phospho-AMPKα (1:1000, Cell Signaling Technology, 2535T, Massachusetts, USA), PGC1-α (1:1000, Protein-tech, 66369-1-Ig, Wuhan, China), NRF-1 (1:1000, Affinity Biosciences, AF5298, Jiangsu, China), NFκB-p65 (1:1000, Affinity Biosciences, AF5006, Jiangsu, China), phospho-NFκB-p65 (1:1000, Affinity Biosciences, AF2006, Jiangsu, China), TNF-α (1:1000, Affinity Biosciences, AF7014, Jiangsu, China), IL-6 (1:2000, Protein-tech, 21865-1-AP, Wuhan, China), IL-1β (1:500, Affinity Biosciences, AF5103, Jiangsu, China), PPAR-γ (1:1000, Affinity Biosciences, AF6284, Jiangsu, China), or phospho-PPAR-γ (1:1000, Affinity Biosciences, AF3284, Jiangsu, China), GLUT-1 (1:5000, Abcam, ab115730, Cambridge, USA) or an antibody against mouse AMPK (1:1000, Affinity Biosciences, AF6423 Jiangsu, China) or GAPDH (1:5000, Affinity Biosciences, AF7021 Jiangsu, China). Additionally, GAPDH was used as a loading control to ensure equal protein loading and transfer.

After washing the membranes thrice with Tris-buffered saline with Tween-20 (TBS/T) for 10 min, they were incubated for 1 h at 25 °C with a secondary antibody (anti-mouse IgG [1:10,000, Affinity Biosciences, BF80002, Jiangsu, China], or anti-rabbit IgG [1:10,000, Affinity Biosciences, S0001, Jiangsu, China]) diluted in 1% BSA-TBS/T. The membranes were washed thrice for 10 min, soaked with Luminol/Enhancer Solution from the Enhanced Chemiluminescence Kit (Thermo Fisher, Shanghai, China), and analyzed using the Chemiluminescence Imaging System (Amersham Imager 680, Cellular Technology Ltd, Monroe, USA). After blotting with the phospho forms of the AMPK, NF-κB, and PPAR-γ antibodies, the membranes were thoroughly stripped. Relative bands, including those of GAPDH, were measured using ImageJ software.

### Quantitative reverse transcription-polymerase chain reaction (RT-PCR) analysis

We used the Trizol reagent (Vazyme, Nanjing, China) to extract total RNA from IMATs (200 μg) isolated from quadriceps of all rats (*n* = 36) in each group. Briefly, IMATs were homogenized with 1 mL Trizol using a mechanical tissue grinder for 3 min and then incubated at 25 °C for 5 min. We then added 200 μL chloroform, thereafter, the samples were shaken vigorously, incubated at 25 °C for 3 min, and centrifuged at 15,000 × *g* for 15 min. The supernatants were transferred to clean tubes, and 500 μL of cold isopropanol was added for RNA precipitation. RNA precipitates were washed with 75% ethanol, dried for 10 min, and re-suspended in RNase-free water. Subsequently, the concentration and purity of each RNA sample was quantified using a Nano50 instrument.

Complementary DNA (cDNA) was synthesized using the PrimeScript™ RT Reagent Kit with gDNA Eraser (Takara, RR047A, Gunma-ken, Japan), and cDNA samples (1.5 μL) were detected by real-time PCR using TB Green ®Premix Ex Taq II (Takara, RR820A, Gunma-ken, Japan). Primers for rat *Ampk*, *Pgc1a*, *Plin5, Il6*, and *Gapdh* mRNA (Table 2) were designed by Sangon Biotech (Shanghai, China). The samples were analyzed using a Light Cycler 480 thermal cycling (Roche, Basel, Switzerland) with the following PCR conditions: denaturation at 95 °C for 15 s, annealing at 60 °C for 30 s, and extension at 72 °C for 30 s, over 40 cycles, following the 2^−Δ∆CT^ method. All gene-expression data were normalized using *Gapdh* mRNA-expression data as an internal control.

**Table 2:** Primer sets used for real-time PCR analysis

Gene	Primers (5′–3′)
*Plin5*-F	GGCTACTTTGTGCGTCTGGGATC
*Plin5*-R	CATCTCCTGGGTGCGGTGTTTG
*Ampk*-F	ATGATGAGGTGGTGGAGCAGAGG
*Ampk*-R	CAGTGAGAGAGCCAGACAGTGAATG
*Pgc1a*-F	CCACTACAGACACCGCACACATC
*Pgc1a*-R	GTATTCGTCCCTCTTCAGCCTTTCG
*Il6*-F	ACTTCCAGCCAGTTGCCTTCTTG
*Il6*-R	TGGTCTGTTGTGGGTGGTATCCTC
*Gapdh*-F	GACATGCCGCCTGGAGAAAC
*Gapdh*-R	AGCCCAGATGCCCTTTAGT

*Plin* perilipin, *Ampk* AMP-activated protein kinase, *F* forward, *Gapdh* glyceraldehyde-3-phosphate dehydrogenase, *Il* interleukin, *Pgc1* peroxisome proliferator-activated receptor-gamma coactivator, *R* reverse

### Statistical analysis

GraphPad Prism Software (v.8.0; GraphPad Software, CA, USA) and SPSS (v.23.0 for Windows; SPSS, Chicago, IL, USA) were used for data analysis, and the results are expressed as the mean ± the standard error of the mean. One-way analysis-of-variance (ANOVA) was used to compare differences between multiple groups for data with homogeneity of variance. For data that did not meet the assumption of homogeneity of variance, nonparametric tests were employed. Two-way repeated measures ANOVA was used to examined the differences between pre-treatment and post-treatment. All ANOVAs were followed by least significant difference (LSD) post hoc tests. Student’s *t*-test was used to analyze two groups. Multiple linear regression was conducted to test associations between two groups and adjust for independent animal effects. Intraclass correlation coefficients (ICCs) with 95% confidence intervals (CIs) for multimodal MRI parameters were calculated for two-way mixed-effects models to assess intra- and inter-reader consistencies. Differences were considered statistically significant at *p* < 0.05.

## Results

### Insulin resistance, impaired glucose tolerance, and moderately increased adiposity in prediabetes

**Fig. 2:**
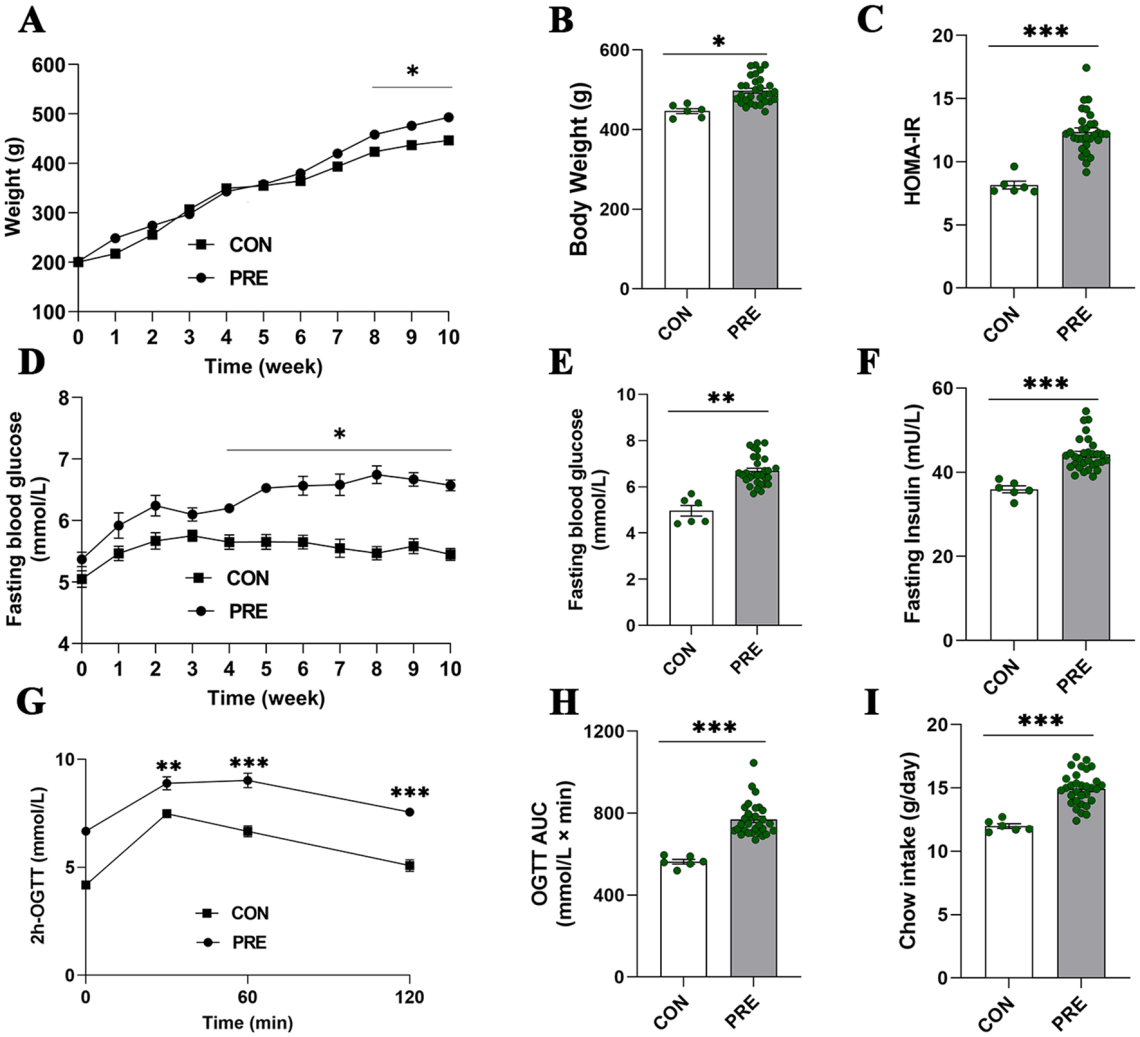
Alterations in body weight and glucose homeostasis indicate the development of prediabetes with high-fat-diet and high-sugar-drink at week 10. **A** Changes in body weights during the experiment. **B** Body-weight data after 10 weeks. **C** Homeostatic model assessment of insulin resistance (HOMA-IR) at week 10. **D**, **E** Fasting-blood glucose levels (**D**) throughout the experiment and at week 10 (**E**). **F**–**H** 2 h oral glucose tolerance test (OGTT) area under the curve (AUC) values (**G**, **H**), fasting-blood insulin levels (**F**), and chow intake results (**I**) at week 10 of the diet. The data shown represent the mean ± standard error of the mean. Control (CON) group, *n* = 6; prediabetic (PRE) group, *n* = 30; Analyses were performed using two-way repeated ANOVA tests for FBG and body weight during week 0–10, and 2 h-OGTT. Student* t*-tests for body weight, HOMA-IR, FBG, FINS, OGTT AUC and Chow intake in week 10 (*p < 0.05, **p < 0.01, ***p < 0.001)

The PRE animals showed significantly higher body weights than the control group starting in week 8, reaching a 7.4% increase in week 10 (*p* < 0.05). FBG levels were elevated in the PRE group by week 4, with a 48% increase by week 10 (*p* < 0.05). By week 10, the 2 h-OGTT and OGTT-AUC levels exhibited impaired glucose tolerance in the PRE group (*p* < 0.01). FINS, and HOMA-IR levels indicated insulin resistance in the PRE group (*p* < 0.001). Additionally, the PRE group displayed significant increase in Chow intake (*p* < 0.001) (Fig. 2).

### Effects of metformin and/or moderate exercise training on body weight, glucose homeostasis, and insulin resistance

Rat body weights were significantly lower in all groups compared with the PRE group (*p* < 0.05). The EMC group lost the most weight (48.07%), followed by the EXE (34.07%) and MET (22.87%) groups, with the EMA group losing the least (18.70%). Moreover, the weight reduction of the MET group was less robust than that of the EMC group (*p* < 0.05), but not the EXE or EMA group. The chow-intake, FBG, FINS, HOMA-IR, and OGTT-AUC values were significantly lower in all groups compared with the PRE group. FBG and FINS levels decreased the most in the EMC group (27.9% and 18.3%, respectively); the HOMA-IR and OGTT-AUC scores in the EMC group decreased by 36.05% and 20.40%, respectively (Additional file 1: Table S1).

### Effects of treatments on adipocyte content and diameter in prediabetic IMAT

**Fig. 3:**
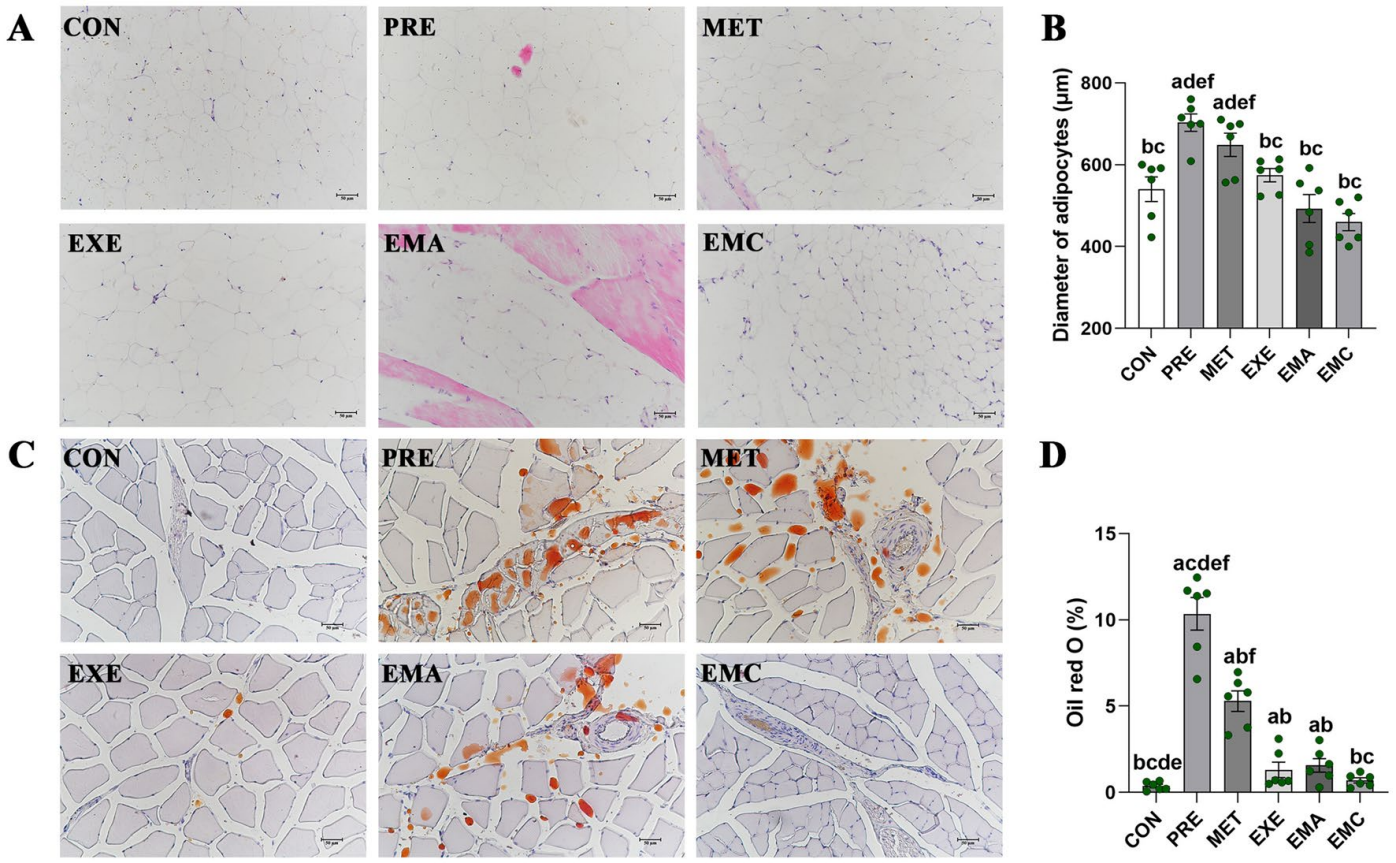
Histological characterization and content of IMAT in muscles. Representative hematoxylin and eosin (H&E) staining and quantification of the diameters of adipocytes in the intermuscular adipose tissue (IMAT) group (**A**, **B**), as well as the representative Oil red O staining and quantification in thigh muscles (**C**, **D**). Magnification: ×200; scale bar = 50 μm. *CON* control, *PRE* prediabetes, *MET* metformin, *EXE* moderate exercise, *EMA* combined therapies + compound-c, *EMC* combined therapies. The data shown are expressed as the mean ± standard error of the mean (n = 6/group). ^a^*p* < 0.05, significantly different from the CON group; ^b^*p* < 0.05, significantly different from the PRE group. ^c^*p* < 0.05, significantly different from the MET group. ^d^*p* < 0.05, significantly different from the EXE group. ^e^*p* < 0.05, significantly different from the EMA group. ^f^*p* < 0.05, significantly different from the EMC group

Metformin treatment alone did not affect adipocyte diameters in IMATs from the MET group (versus the PRE group), whereas IMATs from the EXE, EMA, and EMC groups had smaller-diameter adipocytes than the PRE and MET groups (*p* < 0.05). Moreover, the EMC, EXE, EMA, and control groups had similar IMAT adipocyte diameters (Fig. 3A, B).

Rat skeletal muscle-IMAT levels were lower in the MET, EMA, and EXE (*p* < 0.05) than in the PRE group. However, only the combination treatment decreased the IMAT content to similar levels found in the control group (Fig. 3C, D).

### Effects of exercise and/or metformin on AMPK signaling in IMATs

**Fig. 4:**
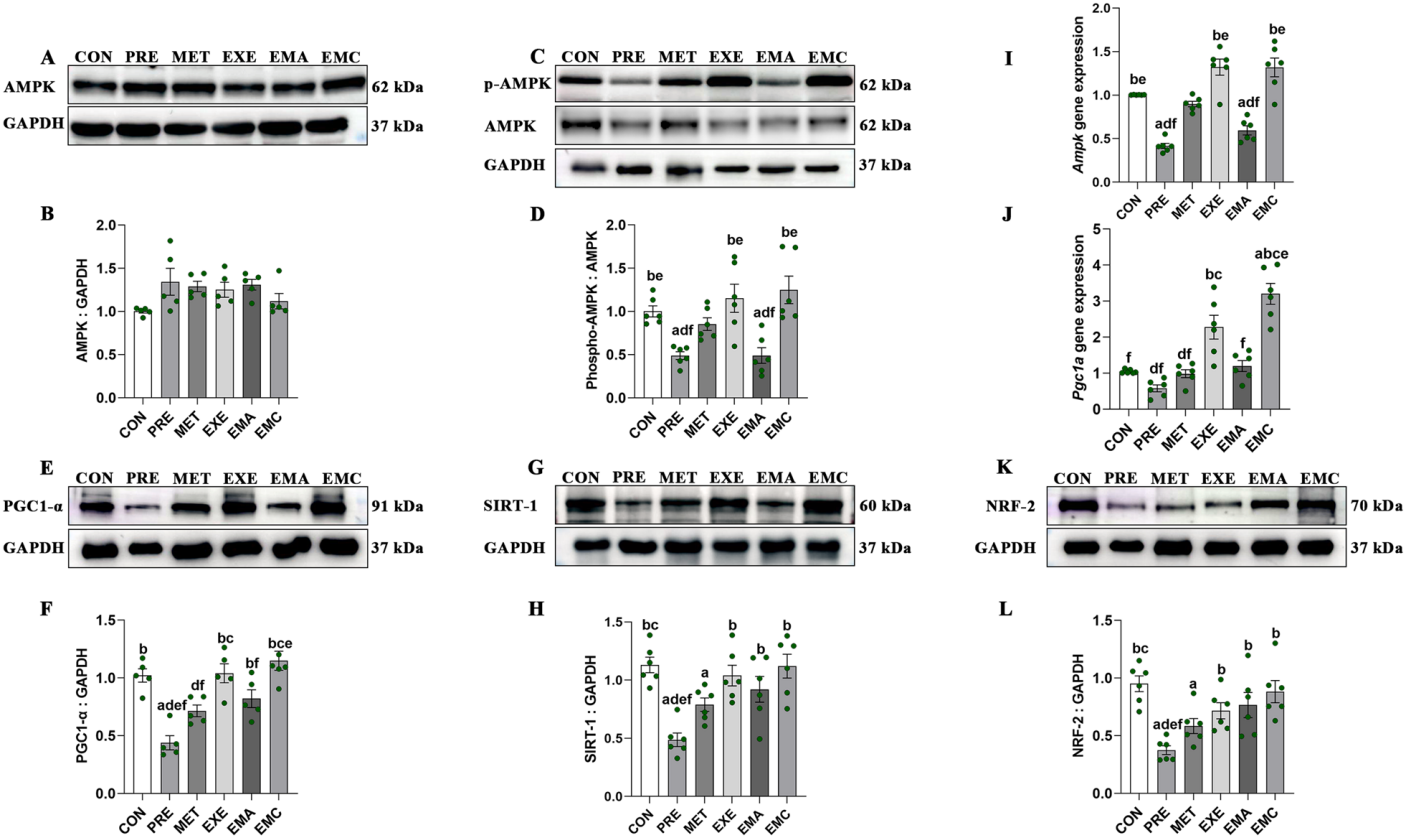
Characterization of AMPK signaling dynamics in intermuscular adipose tissues (IMATs). Representative western blots (**A**, **C**, **E**, **G**, **K**) and quantification of AMP-activated protein kinase (AMPK) and AMPK gene expression (**B**, **I**), phosphorylated AMPK (p-AMPK; Thr172) (**D**), PGC1-α (**F**, **J**), SIRT-1 (**H**) and NRF-2 (**L**). *CON* control, *EMA* combined therapies + compound-c, *EMC* combined therapies, *EXE* moderate-exercise, *GAPDH* glyceraldehyde-3-phosphate dehydrogenase, *MET* metformin, *NRF-2* nuclear factor erythroid-2 related factor 2, *PGC-1α* peroxisome proliferator-activated receptor-γ coactlvator-1α, *PRE* prediabetes, *SIRT-1* silent mating type information regulation 2 homolog-1. The data shown represent mean ± the standard error of the mean (*n* = 5–6/group). ^a^*p* < 0.05 vs. CON group; ^b^*p* < 0.05 vs. PRE group; ^c^*p* < 0.05 vs. MET group; ^d^*p* < 0.05 vs. EXE group; ^e^*p* < 0.05 vs. EMA group; ^f^*p* < 0.05 vs. EMC group

Metformin did not alter the protein expression levels of AMPK or downstream signaling factors in the MET group (Fig. 4A–M). However, the abundances of phospho-AMPK (Thr172), SIRT-1, and NRF-2 were significantly higher in the EXE and EMC groups than in the PRE group (*p* < 0.05). Moreover, phospho-AMPK and *Ampk* expressions were markedly lower in the EMA than in the control group, confirming that compound-c blocked AMPK signaling in the EMA group (Fig. 4C, D). Additionally, the EXE and EMC groups had significantly higher PGC1-α levels than the MET group (*p* < 0.05; Fig. 4E, F). Furthermore, RT-PCR analysis revealed that the expressions of *Ampk* and *Pgc1a* genes were significantly elevated in the EXE and EMC groups compared to the PRE group, while the EXE and EMC groups had higher *Pgc1a* gene expressions compared to the MET group, which aligned with the protein results.

### Effects of treatment on IMAT inflammatory markers in prediabetes

**Fig. 5:**
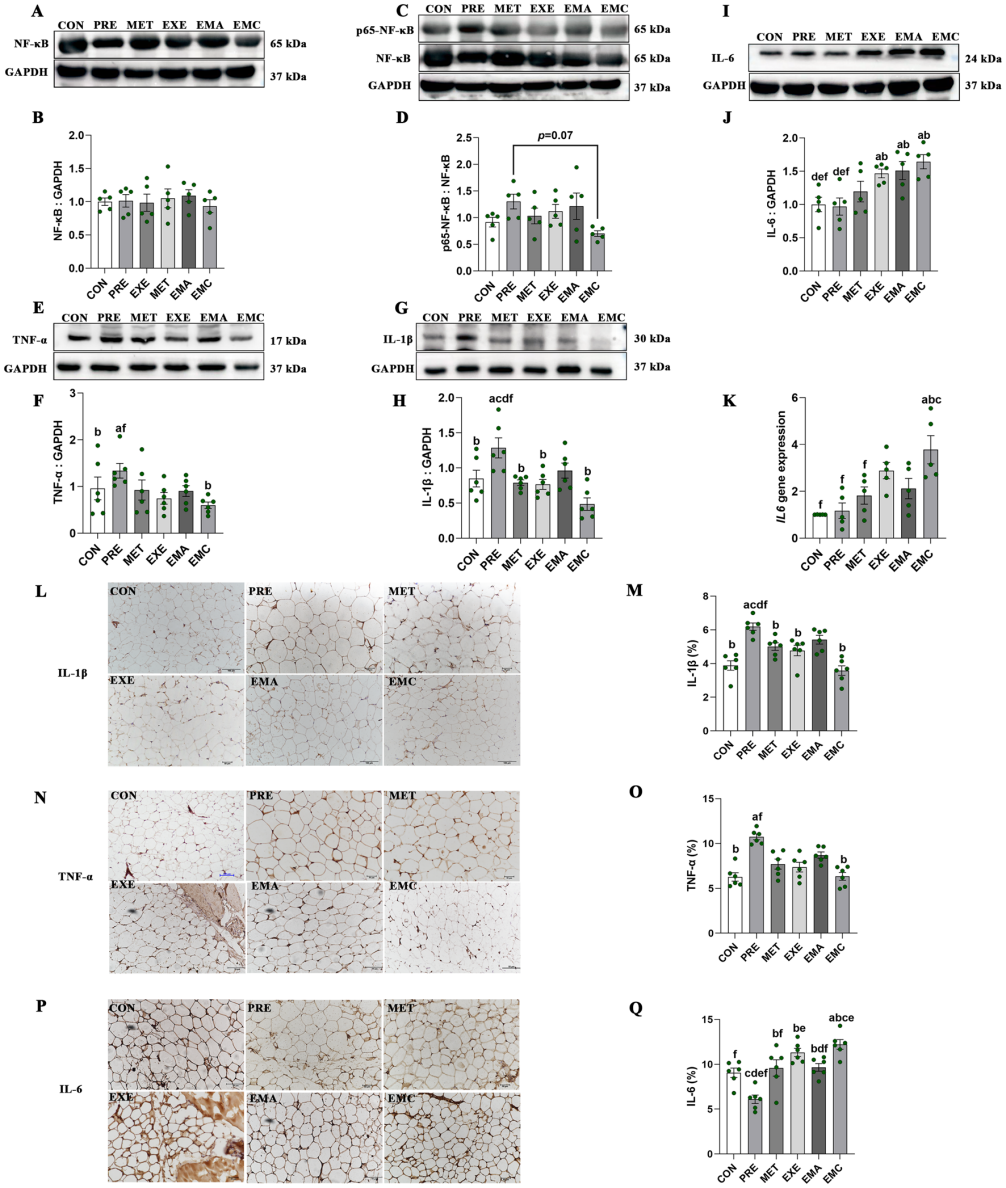
Characterization of inflammation and immune responses in intermuscular adipose tissues (IMATs). Representative western blots (**A**, **C**, **E**, **G**, **I**) and quantification of nuclear factor kappa-B (NF-κB) (**B**), phosphorylated NF-κB (p-NF-κB; p65) (**D**), interleukin-6 (IL-6) (**J**, **K**), tumor necrosis factor-α (TNF-α) (**F**) and interleukin-1β (IL-1β) (**H**). Representative immunostaining and quantification of IL-1β (**L**, **M**), TNF-α (**N**, **O**), and IL-6 (**P**, **Q**) in IMATs. Magnification: ×200; scale bar = 50 μm. *CON* control, *EMA* combined therapies + compound-c, *EMC* combined therapies, *EXE* moderate exercise, *MET* metformin; *PRE* prediabetes. The data shown represent the mean ± standard error of the mean (*n* = 5–6/group). ^a^*p* < 0.05 vs. CON group; ^b^*p* < 0.05 vs.PRE group; ^c^*p* < 0.05 vs. MET group; ^d^*p* < 0.05 vs. EXE group; ^e^*p* < 0.05 vs. EMA group; ^f^*p* < 0.05 vs. EMC group; *p* = 0.07 difference between PRE and EMC groups in terms of the phospho-NF-κB: NF-κB ratio

No significant decrease in NF-κB and phospho-NF-κB (p65) levels was found in the rat IMATs of the EXE, MET, or EMA groups after exercise or metformin treatment (Fig. 5A–D). However, following combination therapy, the IMAT protein content of phospho-NF-κB (p65) was moderately lower in the EMC than in the PRE group (*p* = 0.07, Fig. 5C, D). Furthermore, only the EMC group had significantly lower TNF-α protein levels than the PRE group (*p* < 0.05, Fig. 5E, F). Additionally, excluding the EMA group, all treatment groups effectively reduced IL-1β protein levels compared with the PRE group (*p* < 0.05; Fig. 5G, H).

IL-6 protein abundance was markedly higher in the EXE, EMA, and EMC groups than in the control and PRE groups but not between the MET and control groups (Fig. 5I, J). Additionally, only the EMC group exhibited higher *Il6* expression than the control, PRE, and MET groups (Fig. 5K).

Moreover, immunohistochemical staining revealed that a higher concentration of TNF-α and IL-1β was present in the IMAT cells of prediabetes rats compared to the control group. Meanwhile, the area of IL-1β positivity was significantly reduced in the MET, EXE, and EMC groups relative to the PRE group (Fig. 5L, M). Notably, only the EMC group exhibited significantly lower TNF-α protein content compared to the PRE group (Fig. 5N, O). In addition, all treatment groups exhibited a greater IL-6 positive area than the PRE group. However, only the EMC group had a significantly larger IL-6 positive area than the control, MET, and EMA groups (Fig. 5P, Q).

### Effects of treatment on lipid metabolism-related protein, glucose transporters, and *Plin5* expression in IMATs from prediabetic rats

**Fig. 6:**
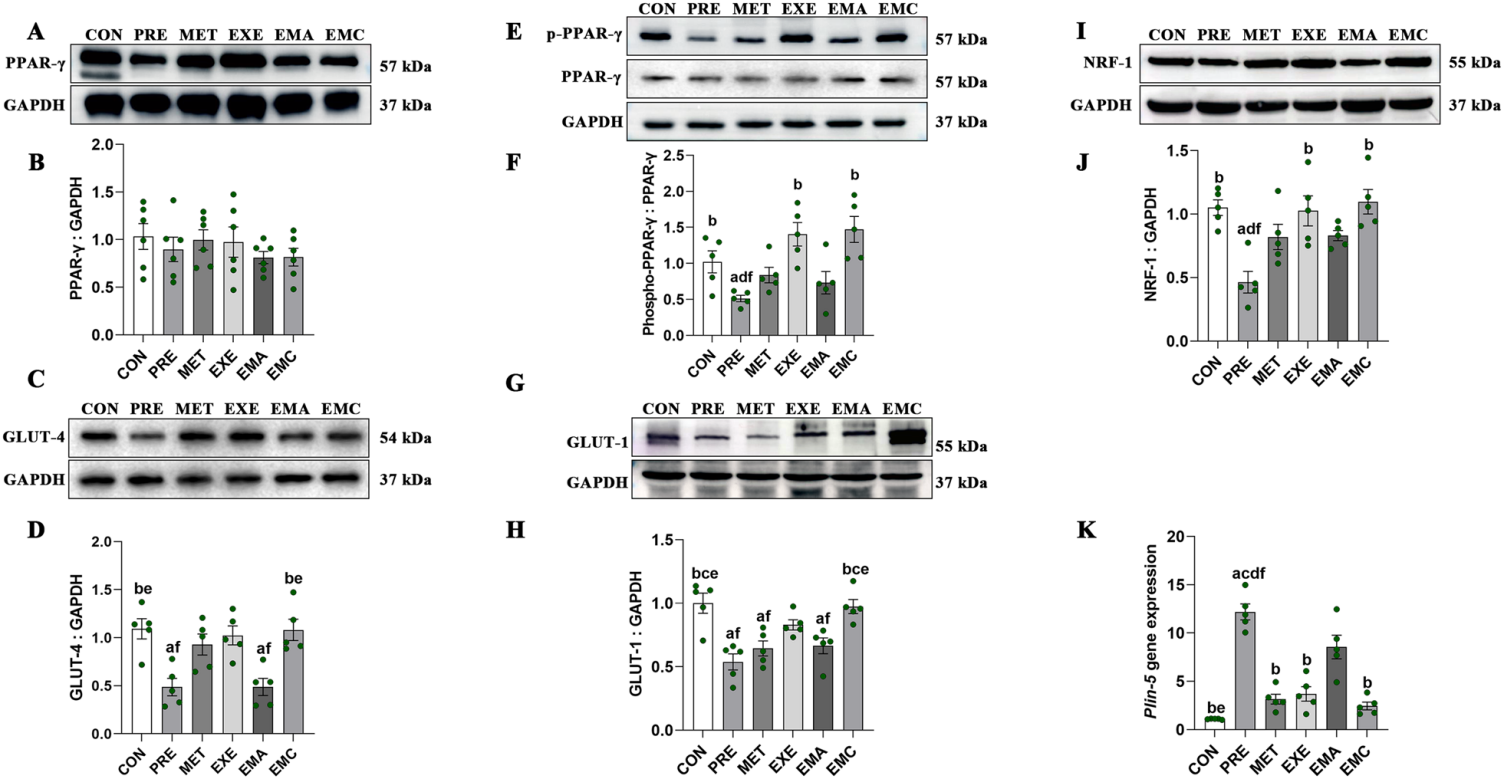
Characterization of lipid and glucose metabolism in intermuscular adipose tissues (IMATs). Representative western blots (**A**, **C**, **E**, **G**, **I**) and quantification of the gene-expression levels of peroxisome proliferators-activated receptor-γ (PPAR-γ) (**B**), phosphorylated PPAR-γ (p-PPAR-γ; Ser112) (**F**), nuclear respiratory factor-1 (NRF-1) (**J**), glucose transporter-4 (GLUT-4) (**D**), glucose transporter-1 (GLUT-1) (**H**), and perilipin-5 (Plin-5) (**K**). The data represent the mean ± the standard error of the mean (*n* = 5–6/group). *CON* control, *EMA* combined therapies + compound-c, *EMC* combined therapies, *EXE* moderate exercise, *GAPDH* glyceraldehyde-3-phosphate dehydrogenase, *MET* metformin, *PRE* prediabetes. ^a^*p* < 0.05 vs. CON group; ^b^*p* < 0.05 vs. PRE group. ^c^*p* < 0.05 vs. MET group; ^d^*p* < 0.05 vs. EXE group; ^e^*p* < 0.05 vs. EMA group; ^f^*p* < 0.05 vs. EMC group

Only the EMC group had significantly higher GLUT-1 and GLUT-4 levels than the PRE group (*p* < 0.05, Fig. 6C, D, G, H). Moreover, significantly higher IMAT phospho-PPAR-γ (Ser112) (*p* < 0.05, Fig. 6E, F) and NRF-1 (*p* < 0.05, Fig. 6I, J) protein levels were found in the EMC and EXE groups than in the PRE group; this was not the case for the MET and EMA groups.

*Plin5* gene expression was significantly lower in all treatment groups (except the EMA group) than in the PRE group (*p* < 0.05; Fig. 6K).

### ICCs for testing the consistency of agreement for multimodal MRI parameters

Intra-reader agreement was high for all measurements (all ICCs > 0.89) and excellent inter-reader reproducibility was observed when assessing IMAT-T2 parameters (ICC, 0.82), IMAT-ADC parameters (ICC, 0.95), IMAT-FA parameters (ICC, 0.96), muscle-T2 parameters, (ICC, 0.93), muscle-ADC parameters (ICC, 0.83), muscle-FA parameters (ICC, 0.86) and the IMAT% (ICC, 0.94) (Table 3).

**Table 3:** Intra- and inter-reader ICC and 95% CI values

Parameter	Intra-reader		Inter-reader	
	ICC	95% CI	ICC	95% CI
*IMAT*-T2	0.91	0.83–0.95	0.82	0.67–0.91
*IMAT*-ADC	0.91	0.85–0.96	0.95	0.91–0.97
*IMAT-*FA	0.96	0.93–0.98	0.96	0.92–0.97
*Muscle*-T2	0.89	0.80–0.94	0.93	0.88–0.96
*Muscle*-ADC	0.91	0.84–0.95	0.83	0.70–0.91
*Muscle*-FA	0.94	0.88–0.97	0.86	0.77–0.93
IMAT%	0.93	0.88–0.96	0.94	0.91–0.97

*ADC* apparent-diffusion coefficient, *CI* confidence interval, *FA* fractional anisotropy, *IMAT* intermuscular adipose tissue, *T2* transverse relaxation time 2

### Multimodal MRI analysis in skeletal muscles and IMATs

The PRE group exhibited higher T2 and ADC values in IMATs and muscles compared to the EXE, EMC, and control groups (*p* < 0.05). T2 and ADC levels in the IMATs were significantly lower in the EMC and EXE groups than in the MET and EMA groups (*p* < 0.05); no significant differences were observed between the MET and EMA groups and the PRE group. Additionally, the EMC and EXE groups showed significantly increased FA values in IMATs compared to the PRE, MET, and EMA groups. However, there was no significant difference in the T2, ADC, or FA levels in the IMATs between the EXE and EMC groups.

**Fig. 7:**
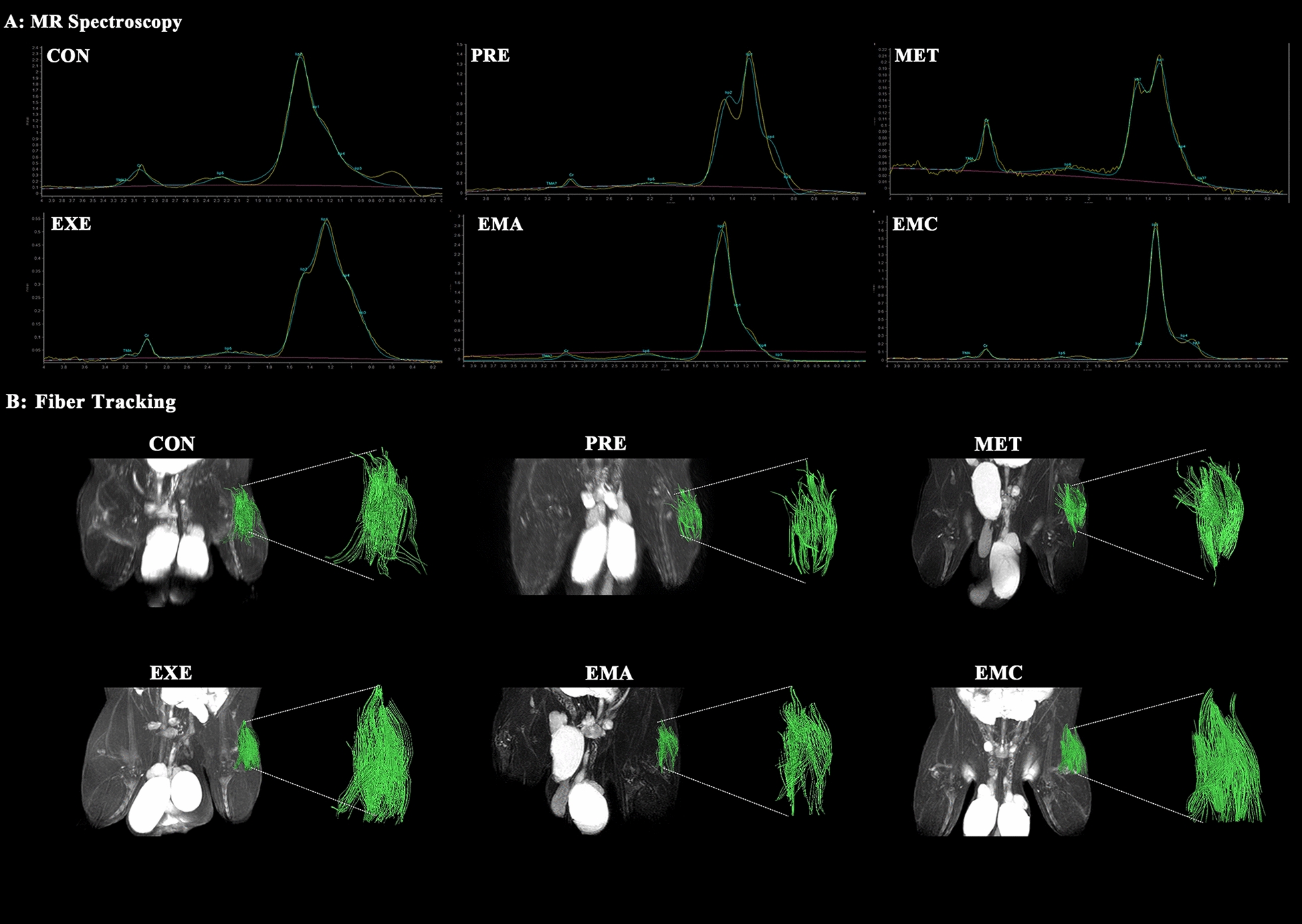
Characterization of representative DTI and MRS images of prediabetic rats. Representative MRS images of the quadriceps femoris in prediabetic rats (**A**); representative schematic diagrams of muscle fiber tracking using DTI in the quadriceps femoris of prediabetic rats (**B**); *CON* control, *EMA* combined therapies + compound-c, *EMC* combined therapies, *EXE* moderate exercise, *MET* metformin, *PRE* prediabetes

In the thigh muscles, the EMC and EXE groups exhibited lower T2, ADC and higher FA levels than the MET and PRE groups (*p* < 0.05). Finally, the IMCL/Cr levels in the EXE, EMA, and EMC treatment groups remained unchanged compared to the PRE and MET groups (see Table 4 at the end of the manuscript and Fig. 7).

**Table 4:** Comparison of muscle and IMAT magnetic-resonance parameters and muscle fat components

Parameter	CON	PRE	MET	EXE	EMA	EMC
IMAT						
T2 (ms)	98.71 ± 2.05^bce^	127.9 ± 1.90^adf^	126.1 ± 1.31^adf^	107.5 ± 3.48^bce^	122.1 ± 2.17^adf^	106.3 ± 1.82^bce^
ADC (10^–3^ mm^2^/s)	0.67 ± 0.03^bce^	0.95 ± 0.09^adf^	0.86 ± 0.02^adf^	0.56 ± 0.02^bce^	0.88 ± 0.05^adf^	0.56 ± 0.03^bce^
FA	0.35 ± 0.02	0.26 ± 0.02^df^	0.28 ± 0.01^df^	0.44 ± 0.03^bce^	0.32 ± 0.04^df^	0.50 ± 0.05^bce^
Muscle						
T2 (ms)	34.26 ± 0.13^b^	37.85 ± 0.37^acdef^	36.19 ± 0.19^bdf^	35.21 ± 0.25^bc^	35.48 ± 0.12^bc^	35.18 ± 0.24^bc^
ADC (10^–3^ mm^2^/s)	1.47 ± 0.02^b^	1.62 ± 0.03^acdf^	1.52 ± 0.02^bdf^	1.41 ± 0.01^bce^	1.56 ± 0.03^bf^	1.43 ± 0.02^bce^
FA	0.26 ± 0.03	0.20 ± 0.01^df^	0.22 ± 0.02^df^	0.30 ± 0.04^bc^	0.23 ± 0.01	0.32 ± 0.03^bc^
IMCL/Cr	8.79 ± 1.57^b^	32.60 ± 9.98^a^	26.58 ± 13.53	16.62 ± 11.68	14.77 ± 4.14	14.92 ± 5.26

Data are expressed as the means ± standard error of the mean (*n* = 6/group)

*ADC* apparent-diffusion coefficient, *CON* control group, *Cr* creatine, *EMA* combined therapies + compound-c group, *EMC* combined therapies group, *EXE* moderate exercise group, *FA* fractional anisotropy, *IMAT* intermuscular adipose tissue, *IMCL* intra-myocellular lipid, *MET* metformin group, *PRE* prediabetes group, *T2* transverse-relaxation time 2

^a^*p* < 0.05, significantly different from the CON. Group

^b^*p* < 0.05, significantly different from the PRE group

^c^*p* < 0.05, significantly different from the MET group

^d^*p* < 0.05, significantly different from the EXE group

^e^*p* < 0.05, significantly different from the EMA group

^f^*p* < 0.05, significantly different from the EMC group

### Effects of treatment on muscle and IMAT cross-sectional areas in prediabetic rats

**Fig. 8:**
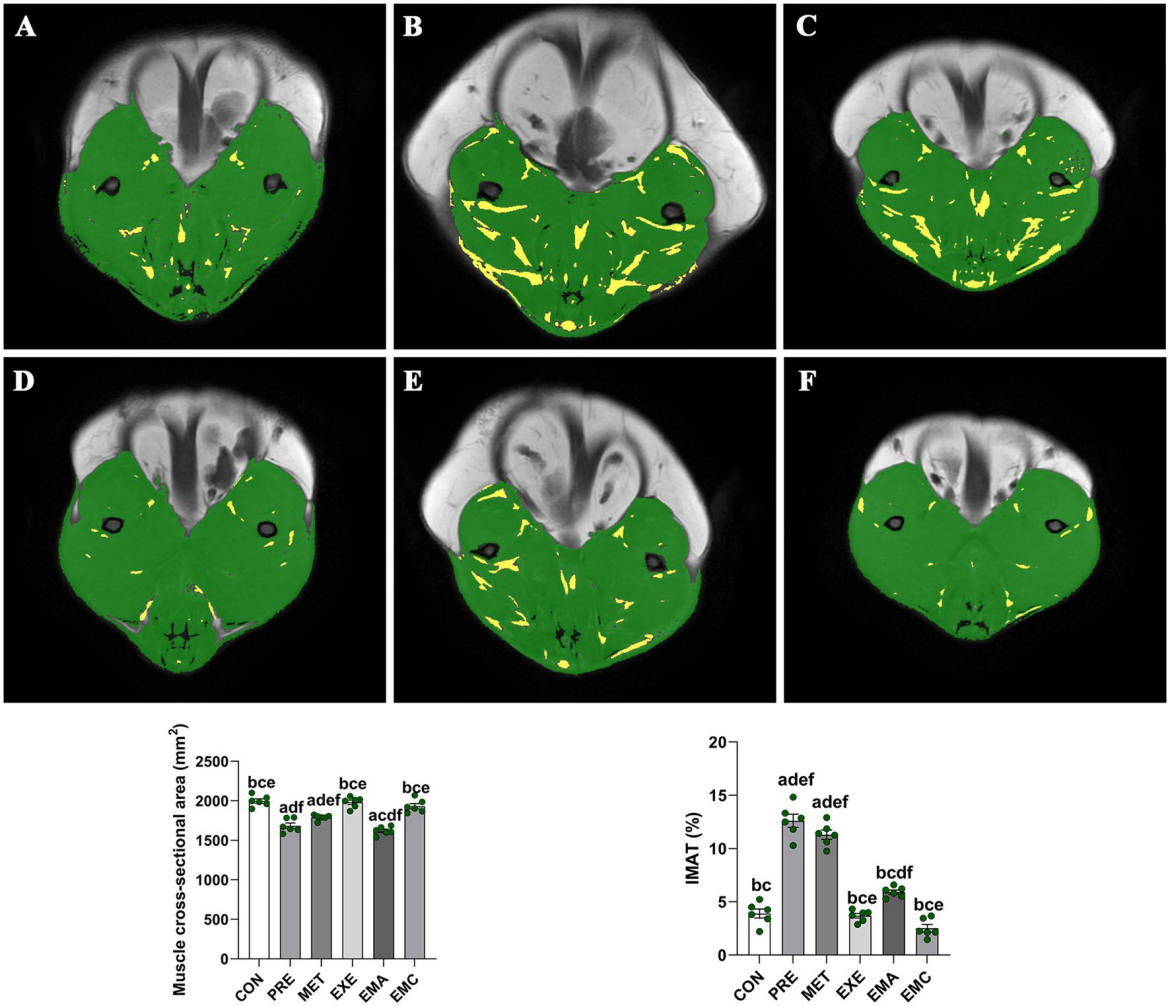
Characterization of the cross-sectional areas of muscle and intermuscular adipose tissues (IMATs) in the middle thigh femur of prediabetic rats. Green regions, skeletal muscles of Wistar rats; yellow regions, IMATs. *CON* control, *EMA* combined therapies + compound-c, *EMC* combined therapies, *EXE* moderate exercise, *MET* metformin, *PRE* prediabetes. The data shown represent the mean ± the standard error of the mean (*n* = 6/group). ^a^*p* < 0.05 vs. CON group; ^b^*p* < 0.05 vs. PRE group; ^c^*p* < 0.05 vs. MET group; ^d^*p* < 0.05 vs. EXE group; ^e^*p* < 0.05 vs. EMA group; ^f^*p* < 0.05, vs. EMC group

The PRE group exhibited higher IMAT% values (*p* < 0.05) than the other treatment and control groups, and had smaller MSCAs than the other groups (*p* < 0.05), with the exception of the MET and EMA groups. The EXE and EMC groups had higher MSCA and lower IMAT% values than (*p* < 0.05) those in the EMA and MET groups; however, the differences were not statistically significant between the EMC or EXE group and the control (Fig. 8).

### Correlations between IMAT magnetic-resonance parameters and laboratory indicators

The T2 and ADC levels in IMATs and muscles, as well as the IMAT (%), were positively associated with FBG, FINS, OGTT-AUC, and HOMA-IR levels (*p* < 0.05). Furthermore, the FA values in IMATs and muscles showed a negative association with FBG, FINS, OGTT-AUC, and HOMA-IR levels. In addition, IMCL/Cr values demonstrated a positive association with FINS (*p* < 0.05) and OGTT-AUC (*p* < 0.05) levels (Table 5).

**Table 5:** Correlations between IMAT magnetic-resonance parameters and laboratory indicators

	FBG		FINS		OGTT-AUC		HOMA-IR	
	*p*	β	*p*	β	*p*	β	*p*	β
IMAT (%)	< 0.01	0.59	< 0.01	0.64	0.02	0.63	< 0.01	0.68
*IMAT*-T2 (ms)	0.03	0.50	0.03	0.47	0.02	0.45	0.01	0.56
*IMAT*-ADC (10^–3^ mm^2^/s)	0.05*	0.51	< 0.01	0.51	0.04	0.40	< 0.01	0.49
*IMAT-*FA	0.04	− 0.45	0.04	− 0.47	0.04	− 0.50	0.02	− 0.51
*Muscle-*T2 (ms)	< 0.01	0.65	< 0.01	0.70	0.02	0.50	< 0.01	0.78
*Muscle-*ADC (10^–3^ mm^2^/s)	0.04	0.35	0.01	0.53	0.05*	0.38	< 0.01	0.60
*Muscle-*FA	0.03	− 0.47	0.05*	− 0.45	0.04	− 0.50	0.01	− 0.53
IMCL/Cr	0.49	0.13	0.01	0.50	0.05*	0.38	0.08	0.32

*ADC* apparent-diffusion coefficient, *Cr* creatine, *FA* fractional anisotropy, *FINS* fasting insulin, *HOMA-IR* homeostatic model assessment of insulin resistance, *IMAT* intermuscular adipose tissue, *IMCL* intra-myocellular lipid, *T2* transverse relaxation time 2

*0.045 < *p* < 0.050

## Discussion

Moderate aerobic exercise and/or metformin effectively treated prediabetic rats, as indicated by lower body weights, FBG, FINS, and OGTT-AUC levels. However, moderate aerobic exercise resulted in higher muscle cross-sectional areas, lower IMAT accumulation in skeletal muscles, smaller adipocyte diameters, increased lipid metabolism and mitochondrial activity, and decreased inflammation in IMATs. Modestly lower TNF-α and phospho-NF-κB levels and higher GLUT-1,4 levels were found in IMATs from prediabetic rats administered combined treatment. Multimodal MRI data confirmed that combined treatment offered minimal additional benefit against prediabetes, and metformin alone was less effective against prediabetes than aerobic exercise. However, metformin did not counterbalance the beneficial effects of exercise.

IMAT is associated with T2DM [19, 28, 29] and may help predict T2DM [28]. The prediabetic rats had more IMAT than the control group, and the IMAT contents of thigh muscles correlated positively with FBG, FINS, and OGTT-AUC levels. IMAT has also been associated with IR in prediabetes [28]. Therefore, a combination of effective medications and lifestyle modifications (e.g., drug and exercise) is needed to lower IMAT contents.

As previously mentioned, both moderate aerobic exercise and metformin have been demonstrated to be beneficial for improving metabolism in skeletal muscles. However, their individual or combined application in IMAT remains unknown. Aerobic exercise primarily modulated AMPK, SIRT-1, and PGC1-α pathways to increase energy substrate availability and prevent IR [30]. Similarly, our results suggested that exercise alone activated the phospho-AMPK in IMATs, leading to an increase in the abundance of PGC-1α, SIRT-1 and NRF-2, which in turn promoted cellular antioxidant responses in IMAT. Also, we found decreased IL-1β, smaller diameters of IMAT adipocytes and lower IMAT contents in exercise only group, suggesting moderate aerobic exercise can reduce the inflammation and improve adipose metabolism in IMAT effectively. PPAR-γ and downstream proteins could effectively protect non-adipose tissues and organs against excessive lipid overload [31]. For instance, *Plin5* expression could promote lipolysis in IMAT in patients with insulin resistance [5]. We found that phospho-PPAR-γ levels were significantly higher in the EXE and EMC groups than in the PRE group, suggesting that combined therapy and exercise alone can improve insulin sensitivity and diabetes by targeting the PPAR-γ-signaling pathway. Furthermore, *Plin5* downregulation and NRF-1 upregulation suggested that exercise alone can promote lipolysis and boost fat oxidation in IMATs. Thus, during prediabetes, high free-fatty acid levels produced by lipolysis in adipose tissues promoted lipotoxicity and insulin resistance [32], whereas exercise promoted lipolysis and directly oxidized IMAT resulting in fat reduction in thigh muscles. Of interest, only EMC group showed increased IL-6 gene expression compared with PRE and MET groups. Previous studies have shown that IL-6 has varied roles in different human organs and can have both pro-inflammatory and anti-inflammatory effects [33]. During exercise, IL-6 stimulates fat breakdown and enhances insulin sensitivity by promoting an anti-inflammatory environment and facilitating the translocation of GLUT-1 and GLUT-4 to the plasma membrane [33]. Of note, our findings showed that, the combined therapy group had markedly elevated levels of GLUT-1 and GLUT-4 compared to the PRE and MET groups, whereas the EXE group had no significant elevation. Also, only combined therapy reduced the levels of phosphor-NF-κB and TNF-α compared to the PRE group. Furthermore, Compound-c also blocked AMPK activation in the EMA group, further suggesting that combination therapy might affect IMAT metabolism in prediabetes through the AMPK-signaling pathway. Our results suggest that combined therapy may offer some additional benefits in terms of inflammation and glucose metabolism within IMATs.

Unlike moderate-intensity exercise, metformin might reduce the positive effects of exercise on insulin sensitivity and AMPK activity in skeletal muscles [15]. Further, metformin impeded mitochondrial respiratory-chain complex 1 [34], and might, thus, block energy needed for aerobic exercise. whereas metformin treatment alone was less effective. In humans, metformin is primarily transported to skeletal muscle cells via organic cation transport protein-3 [35]. Previous data showed that the actual metformin concentration in skeletal muscles was significantly lower than the pharmacological concentration in patients’ plasma [36]. In contrast, in vitro and animal data using clinically achievable metformin concentrations (< 100 µM) do not show negative effects on mitochondria [37, 38]. Therefore, whether clinically pharmacological doses of metformin can reach the concentration thresholds in skeletal muscles or IMATs necessary to influence the effects of aerobic exercise is unclear [39]. Our results showed that metformin did not significantly increase energy metabolism in IMAT mitochondria or inhibit the therapeutic effect of moderate aerobic exercise in the combination treatments. Moreover, larger MSCAs represent a critical factor that affects muscle strength [40]. MSCAs can also mediate the link between a higher percentage of IMAT and impaired physical function [41]. Here, we found that metformin treatment alone did not effectively recover the cross-sectional muscle area caused by prediabetes or decrease the IMAT% in muscles. In contrast, combination therapy and aerobic exercise alone effectively recovered MSCAs and reduced the IMAT%. The average MSCA value associated with aerobic exercise therapy was approximately 4.9% higher than that of the combined therapy group, indicating that adding metformin to aerobic exercise may not increase muscle strength. Overall, our findings suggest that metformin does not seem to offset the positive impact of exercise on IMAT in subjects with prediabetes through AMPK pathways. Thus, further studies are needed to investigate whether metformin and aerobic exercise interact in other tissues where metformin is primarily absorbed and metabolized (e.g., the intestines).

Although a close association exists between skeletal-muscle MR parameters, IR, and T2DM [42, 43], few studies have investigated the correlation between IMAT and MR parameters in prediabetes. Research confirmed that, T2 and ADC levels were significantly higher in patients with inflammatory edema, while FA levels were significantly lower, compared to healthy subjects [44, 45]. In our study, we found that T2, ADC, FA, and IMAT (%) values correlated positively with key clinical indicators (fasting blood glucose, fasting insulin, OGTT-AUC, and HOMA-IR) in IMAT and muscles, indicating that multimodal MRI can help detect metabolic changes in IMATs in prediabetes subjects.

Moreover, T2 values tended to increase with the severity of muscle fat or inflammatory cell infiltration, muscle edema, and fat infiltration [46]. ADC and FA values, on the other hand, enable quantitative analysis of water diffusion in tissues, which is closely related to the structure and function of the tissue microenvironment. Therefore, when evaluating metabolic changes in skeletal muscles, T2 mapping can reflect the stage of prediabetes, whereas DTI sequences reveal metabolic alterations in skeletal muscles during treatment. In the present study, we found decreased T2, ADC, as well as improved FA values in EMC and EXE groups compared to MET and EMA groups, with both groups exhibiting significantly inIMATs and muscles. Consequently, both exercise and combined therapy improved IMAT and muscle functions, which aligned with the histological results.

The IMCL contents in skeletal muscles did not differ between rats in the EMC, EXE, and EMA groups compared with those in the PRE and MET groups. Our findings align with those of Sabine et al., who found that exercise training improved insulin sensitivity in patients with T2DM, without affecting the total IMCL content [47]. This discrepancy may be explained by the ‘athlete’s paradox’, where both athletes and T2D patients show high IMCL levels [48]. Previous findings suggest that IMCLs did not influence IR but might increase with improved coordination between the fuel supply (e.g., triglycerides) and energetic capacity during exercise [49].

**Fig. 9:**
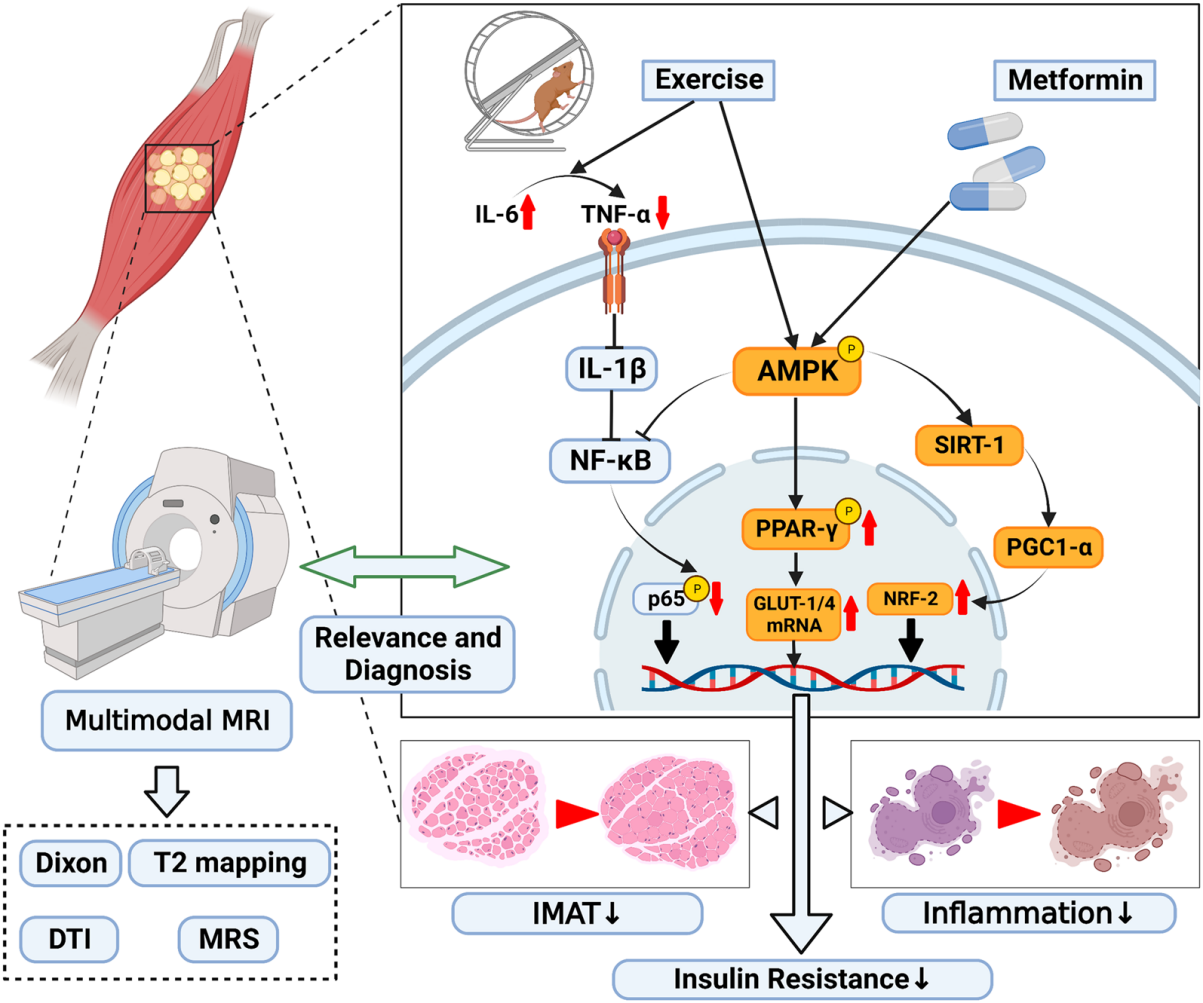
Potential mechanisms whereby moderate aerobic exercise, metformin alone, or combined intervention affects IMATs in prediabetic rats (created by BioRender). *AMPK* AMP-activated protein kinase, *DTI* diffusion-tensor imaging, *GLUT-1* glucose transporter-1, *GLUT-4* glucose transporter-4, *IL-6* interleukin-6, *IMAT* intermuscular adipose tissue, *MRI* magnetic-resonance imaging, *MRS* magnetic-resonance spectroscopy, *NF-κB* nuclear factor kappa-B, *NRF-2* nuclear respiratory factor-2, *PGC1-α* peroxisome proliferator-activated receptor-γ coactlvator-1α, *PPAR-γ* peroxisome proliferators-activated receptor-γ, *SIRT-1* silent mating type information regulation 2 homolog-1, *TNF-α* tumor necrosis factor-α

Overall, our results indicate that moderate aerobic exercise was an effective lifestyle intervention for subjects with prediabetes. Moderate aerobic exercise restored muscle strength, reduced IMAT levels, decreased inflammation, and restored oxidative metabolism via AMPK activation. In contrast, metformin had less significant effects on IMAT levels and muscle metabolism. Adding metformin to aerobic exercise did not appear to offer significant synergistic effects. Thus, whether metformin should be used with exercise in managing prediabetes needs to be further evaluated. A better understanding of interactions between metformin and exercise could help maximize their combined benefits (Fig. 9).

This study has several limitations. First, we did not directly observe the morphology of mitochondria in IMATs or analyze respiratory chain complex-1 in mitochondria. Second, we did not directly measure specific metformin concentrations in skeletal muscles or IMATs in prediabetic rats. Third, we did not subject the extracted IMATs to primary cell culture to continue validating our findings at the cellular level. Thus, future studies should examine the influence of AMPK- and mitochondria-related signaling pathways on other specific genes and affected cellular populations within treated muscles.

## Conclusion

Moderate aerobic exercise training alone effectively inhibits IMAT function and structure via the AMPK pathway in prediabetic rats. Regarding IMAT-metabolism and multimodal MRI parameters, metformin is not as effective alone or in combination. Hence, while combining metformin with moderate aerobic exercise might elicit some modest synergy, it does not appear to counterbalance the beneficial effects of exercise.

### Supplementary Information

**Additional file 1: Table S1.** Body-weight, fasting-plasma insulin, glucose, OGTT-AUC, and HOMA-IR values before and after high-fat diet feeding.

## Data Availability

The original data generated in this study are available at Mendeley Data (10.17632/35mkyzcc7p.1).

## Abbreviations

IMAT: Intermuscular adipose tissue
T2DM: Type 2 diabetes
AMPK: Adenosine 5′-monophosphate-activated protein kinase
PGC1-α: Peroxisome proliferator-activated receptor-γ coactlvator-1α
NRF-2: Nuclear factor erythroid 2-related factor
NF-κB: Nuclear factor-kappa B
TNF-⍺: Tumor necrosis factor alpha
IL-1β: Interleukin-1 beta
IL-6: Interleukin-6
AUC: Area under the curve
PPAR-γ: Peroxisome proliferators-activated receptor-gamma
GLUT: Glucose transporter
NRF-1: Nuclear respiratory factor-1
GAPDH: Glyceraldehyde-3-phosphate dehydrogenase
FBG: Fasting blood glucose
OGTT: Oral glucose tolerance test
HOMA: Homeostasis model assessment
IR: Insulin resistance
STZ: Streptozotocin
MRI: Magnetic resonance imaging
ADC: Apparent diffusion coefficient
FA: Fractional anisotropy
MRS: Magnetic resonance spectroscopy
IMCL: Intramyocellular lipids
DTI: Diffusion tensor imaging
Cr: Creatinine
ROI: Regions of interest
MSCA: Muscle cross-sectional area

## References

[1] Schlesinger S, Neuenschwander M, Barbaresko J, Lang A, Maalmi H, Rathmann W, Roden M, Herder C (2022). Prediabetes and risk of mortality, diabetes-related complications and comorbidities: umbrella review of meta-analyses of prospective studies. Diabetologia.

[2] Chai Y, Liu Y, Yang R, Kuang M, Qiu J, Zou Y (2022). Association of body mass index with risk of prediabetes in Chinese adults: a population-based cohort study. J Diabetes Investig.

[3] Goodpaster BH, Bergman BC, Brennan AM, Sparks LM (2023). Intermuscular adipose tissue in metabolic disease. Nat Rev Endocrinol.

[4] Miljkovic I, Cauley JA, Wang PY, Holton KF, Lee CG, Sheu Y, Barrett-Connor E, Hoffman AR, Lewis CB, Orwoll ES, Stefanick ML, Strotmeyer ES, Marshall LM (2013). Abdominal myosteatosis is independently associated with hyperinsulinemia and insulin resistance among older men without diabetes. Obesity (Silver Spring).

[5] Sachs S, Zarini S, Kahn DE, Harrison KA, Perreault L, Phang T, Newsom SA, Strauss A, Kerege A, Schoen JA, Bessesen DH, Schwarzmayr T, Graf E, Lutter D, Krumsiek J, Hofmann SM, Bergman BC (2019). Intermuscular adipose tissue directly modulates skeletal muscle insulin sensitivity in humans. Am J Physiol Endocrinol Metab.

[6] Kahn D, Macias E, Zarini S, Garfield A, Zemski Berry K, Gerszten R, Schoen J, Cree-Green M, Bergman BC (2022). Quantifying the inflammatory secretome of human intermuscular adipose tissue. Physiol Rep.

[7] Færch K, Amadid H, Nielsen LB, Ried-Larsen M, Karstoft K, Persson F, Jørgensen ME (2017). Protocol for a randomised controlled trial of the effect of dapagliflozin, metformin and exercise on glycaemic variability, body composition and cardiovascular risk in prediabetes (the PRE-D trial). BMJ Open.

[8] Draznin B, Aroda VR, Bakris G, Benson G, Brown FM, Freeman R, Green J, Huang E, Isaacs D, Kahan S, Leon J, Lyons SK, Peters AL, Prahalad P, Reusch JEB, Young-Hyman D (2022). 3. Prevention or delay of type 2 diabetes and associated comorbidities: standards of medical care in diabetes-2022. Diabetes Care.

[9] Fujita Y, Inagaki N (2017). Metformin: new preparations and nonglycemic benefits. Curr Diab Rep.

[10] Rena G, Hardie DG, Pearson ER (2017). The mechanisms of action of metformin. Diabetologia.

[11] Hostalek U, Gwilt M, Hildemann S (2015). Therapeutic use of metformin in prediabetes and diabetes prevention. Drugs.

[12] Kjøbsted R, Munk-Hansen N, Birk JB, Foretz M, Viollet B, Björnholm M, Zierath JR, Treebak JT, Wojtaszewski JF (2017). Enhanced muscle insulin sensitivity after contraction/exercise is mediated by AMPK. Diabetes.

[13] Hayashi T, Wojtaszewski JF, Goodyear LJ (1997). Exercise regulation of glucose transport in skeletal muscle. Am J Physiol.

[14] Jevtovic F (2021). Combination of metformin and exercise in management of metabolic abnormalities observed in type 2 diabetes mellitus. Diabetes Metab Syndr Obes.

[15] Sharoff CG, Hagobian TA, Malin SK, Chipkin SR, Yu H, Hirshman MF, Goodyear LJ, Braun B (2010). Combining short-term metformin treatment and one bout of exercise does not increase insulin action in insulin-resistant individuals. Am J Physiol Endocrinol Metab.

[16] Ortega JF, Morales-Palomo F, Ramirez-Jimenez M, Moreno-Cabañas A, Mora-Rodríguez R (2020). Exercise improves metformin 72-h glucose control by reducing the frequency of hyperglycemic peaks. Acta Diabetol.

[17] Richter EA, Ruderman NB (2009). AMPK and the biochemistry of exercise: implications for human health and disease. Biochem J.

[18] Ma T, Tian X, Zhang B, Li M, Wang Y, Yang C, Wu J, Wei X, Qu Q, Yu Y, Long S, Feng JW, Li C, Zhang C, Xie C, Wu Y, Xu Z, Chen J, Yu Y, Huang X, He Y, Yao L, Zhang L, Zhu M, Wang W, Wang ZC, Zhang M, Bao Y, Jia W, Lin SY, Ye Z, Piao HL, Deng X, Zhang CS, Lin SC (2022). Low-dose metformin targets the lysosomal AMPK pathway through PEN2. Nature.

[19] Yu F, He B, Chen L, Wang F, Zhu H, Dong Y, Pan S (2021). Intermuscular fat content in young Chinese men with newly diagnosed type 2 diabetes: based on MR mDIXON-quant quantitative technique. Front Endocrinol (Lausanne).

[20] Hooijmans MT, Damon BM, Froeling M, Versluis MJ, Burakiewicz J, Verschuuren JJ, Niks EH, Webb AG, Kan HE (2015). Evaluation of skeletal muscle DTI in patients with duchenne muscular dystrophy. NMR Biomed.

[21] Koncsos G, Varga ZV, Baranyai T, Boengler K, Rohrbach S, Li L, Schlüter KD, Schreckenberg R, Radovits T, Oláh A, Mátyás C, Lux Á, Al-Khrasani M, Komlódi T, Bukosza N, Máthé D, Deres L, Barteková M, Rajtík T, Adameová A, Szigeti K, Hamar P, Helyes Z, Tretter L, Pacher P, Merkely B, Giricz Z, Schulz R, Ferdinandy P (2016). Diastolic dysfunction in prediabetic male rats: role of mitochondrial oxidative stress. Am J Physiol Heart Circ Physiol.

[22] Najafipour H, Rostamzadeh F, Yeganeh-Hajahmadi M, Joukar S (2021). Improvement of cardiac function in rats with myocardial infarction by low-intensity to moderate-intensity endurance exercise is associated with normalization of Klotho and SIRT1. J Cardiovasc Pharmacol.

[23] Liu J, Lu J, Zhang L, Liu Y, Zhang Y, Gao Y, Yuan X, Xiang M, Tang Q (2023). The combination of exercise and metformin inhibits TGF-β1/Smad pathway to attenuate myocardial fibrosis in db/db mice by reducing NF-κB-mediated inflammatory response. Biomed Pharmacother.

[24] Bullón P, Alcocer-Gómez E, Carrión AM, Marín-Aguilar F, Garrido-Maraver J, Román-Malo L, Ruiz-Cabello J, Culic O, Ryffel B, Apetoh L, Ghiringhelli F, Battino M, Sánchez-Alcazar JA, Cordero MD (2016). AMPK phosphorylation modulates pain by activation of NLRP3 inflammasome. Antioxid Redox Signal.

[25] Jagomäe T, Seppa K, Reimets R, Pastak M, Plaas M, Hickey MA, Kukker KG, Moons L, De Groef L, Vasar E, Kaasik A, Terasmaa A, Plaas M (2021). Early intervention and lifelong treatment with GLP1 receptor agonist liraglutide in a wolfram syndrome rat model with an emphasis on visual neurodegeneration, sensorineural hearing loss and diabetic phenotype. Cells.

[26] Yao L, Wang C, Zhang X, Peng L, Liu W, Zhang X, Liu Y, He J, Jiang C, Ai D, Zhu Y (2016). Hyperhomocysteinemia activates the aryl hydrocarbon receptor/CD36 pathway to promote hepatic steatosis in mice. Hepatology.

[27] Li L, Wang H, Zhang J, Chen X, Zhang Z, Li Q (2021). Effect of endothelial progenitor cell-derived extracellular vesicles on endothelial cell ferroptosis and atherosclerotic vascular endothelial injury. Cell Death Discov.

[28] Granados A, Gebremariam A, Gidding SS, Terry JG, Carr JJ, Steffen LM, Jacobs DR, Lee JM (2019). Association of abdominal muscle composition with prediabetes and diabetes: the CARDIA study. Diabetes Obes Metab.

[29] Goss AM, Gower BA (2012). Insulin sensitivity is associated with thigh adipose tissue distribution in healthy postmenopausal women. Metabolism.

[30] Vogt ÉL, Von Dentz MC, Rocha DS, Model JFA, Kowalewski LS, Silveira D, de Amaral M, de Bittencourt Júnior PIH, Kucharski LC, Krause M, Vinagre AS (2023). Acute effects of a single moderate-intensity exercise bout performed in fast or fed states on cell metabolism and signaling: comparison between lean and obese rats. Life Sci.

[31] Kintscher U, Law RE (2005). PPARgamma-mediated insulin sensitization: the importance of fat versus muscle. Am J Physiol Endocrinol Metab.

[32] Boden G, Shulman GI (2002). Free fatty acids in obesity and type 2 diabetes: defining their role in the development of insulin resistance and beta-cell dysfunction. Eur J Clin Invest.

[33] Akbari M, Hassan-Zadeh V (2018). IL-6 signalling pathways and the development of type 2 diabetes. Inflammopharmacology.

[34] Owen MR, Doran E, Halestrap AP (2000). Evidence that metformin exerts its anti-diabetic effects through inhibition of complex 1 of the mitochondrial respiratory chain. Biochem J.

[35] Gong L, Goswami S, Giacomini KM, Altman RB, Klein TE (2012). Metformin pathways: pharmacokinetics and pharmacodynamics. Pharmacogenet Genom.

[36] Gormsen LC, Sundelin EI, Jensen JB, Vendelbo MH, Jakobsen S, Munk OL, Hougaard Christensen MM, Brøsen K, Frøkiær J, Jessen N (2016). In vivo imaging of human 11C-metformin in peripheral organs: dosimetry, biodistribution, and kinetic analyses. J Nucl Med.

[37] Fontaine E (2018). Metformin-induced mitochondrial complex I inhibition: facts, uncertainties, and consequences. Front Endocrinol (Lausanne).

[38] Wang Y, An H, Liu T, Qin C, Sesaki H, Guo S, Radovick S, Hussain M, Maheshwari A, Wondisford FE, O'Rourke B, He L (2019). Metformin improves mitochondrial respiratory activity through activation of AMPK. Cell Rep.

[39] Kristensen JM, Lillelund C, Kjøbsted R, Birk JB, Andersen NR, Nybo L, Mellberg K, Balendran A, Richter EA, Wojtaszewski JFP (2019). Metformin does not compromise energy status in human skeletal muscle at rest or during acute exercise: a randomised, crossover trial. Physiol Rep.

[40] Suchomel TJ, Nimphius S, Bellon CR, Stone MH (2018). The importance of muscular strength: training considerations. Sports Med.

[41] Farsijani S, Santanasto AJ, Miljkovic I, Boudreau RM, Goodpaster BH, Kritchevsky SB, Newman AB (2021). The relationship between intermuscular fat and physical performance is moderated by muscle area in older adults. J Gerontol A Biol Sci Med Sci.

[42] Boersma GJ, Johansson E, Pereira MJ, Heurling K, Skrtic S, Lau J, Katsogiannos P, Panagiotou G, Lubberink M, Kullberg J, Ahlström H, Eriksson JW (2018). Altered glucose uptake in muscle, visceral adipose tissue, and brain predict whole-body insulin resistance and may contribute to the development of type 2 diabetes: a combined PET/MR study. Horm Metab Res.

[43] Ferrannini E, Iozzo P, Virtanen KA, Honka MJ, Bucci M, Nuutila P (2018). Adipose tissue and skeletal muscle insulin-mediated glucose uptake in insulin resistance: role of blood flow and diabetes. Am J Clin Nutr.

[44] Qi J, Olsen NJ, Price RR, Winston JA, Park JH (2008). Diffusion-weighted imaging of inflammatory myopathies: polymyositis and dermatomyositis. J Magn Reson Imaging.

[45] Willcocks R (2014). Global T2 versus water T2 in NMR imaging of fatty infiltrated muscles: different methodology, different information, and different implications. Neuromuscul Disord.

[46] Huang R, Yang H, Chen L, Su S, Wu X, Zhuang R, Liu Y (2022). T2 mapping and fat quantification of lumbar paraspinal muscle in ankylosing spondylitis: a case control study. BMC Musculoskelet Disord.

[47] Daemen S, Gemmink A, Brouwers B, Meex RCR, Huntjens PR, Schaart G, Moonen-Kornips E, Jörgensen J, Hoeks J, Schrauwen P, Hesselink MKC (2018). Distinct lipid droplet characteristics and distribution unmask the apparent contradiction of the athlete’s paradox. Mol Metab.

[48] Gemmink A, Daemen S, Brouwers B, Hoeks J, Schaart G, Knoops K, Schrauwen P, Hesselink MKC (2021). Decoration of myocellular lipid droplets with perilipins as a marker for in vivo lipid droplet dynamics: a super-resolution microscopy study in trained athletes and insulin resistant individuals. Biochim Biophys Acta Mol Cell Biol Lipids.

[49] Dubé JJ, Amati F, Stefanovic-Racic M, Toledo FG, Sauers SE, Goodpaster BH (2008). Exercise-induced alterations in intramyocellular lipids and insulin resistance: the athlete’s paradox revisited. Am J Physiol Endocrinol Metab.

