# Supplementary material for: Aerobic exercise and metformin on intermuscular adipose tissue (IMAT): insights from multimodal MRI and histological changes in prediabetic rats
Source: Diabetol Metab Syndr. 2023 Oct 30;15:221. doi: 10.1186/s13098-023-01183-x (PMC10614363; doi:10.1186/s13098-023-01183-x)
Supplement: Supplementary file 1 — Additional file 1: Table S1. Body-weight, fasting-plasma insulin, glucose, OGTT-AUC, and HOMA-IR values before and after high-fat diet feeding. [file 13098_2023_1183_MOESM1_ESM.docx]

Table S1. Body-weight, fasting-plasma insulin, glucose, OGTT-AUC, and HOMA-IR values before and after high-fat diet feeding

|  | CON | PRE | MET | EXE | EMA | EMC |
| --- | --- | --- | --- | --- | --- | --- |
| *n* | 6 | 6 | 6 | 6 | 6 | 6 |
| Body weight (g) | | | | | | |
| *Week 10* | 446.4 ± 8.0^bcdef^ | 485.8 ± 1.7^a^ | 501.4 ± 16.3^a^ | 496.2 ± 12.3^a^ | 493.0 ± 6.7^a^ | 498.8 ± 6.4^a^ |
| *Week 14* | 497.4 ± 7.5^bcdef^ | 573.6 ± 25.7^acdef^ | 386.7 ± 15.4^abf*^ | 324.0 ± 13.6^abe*^ | 400.8 ± 10.1^abdf*^ | 259.0 ± 13.4^abce*^ |
| Chow intake (g) | | | | | | |
| *Week 10* | 12.2 ± 0.3^bcdef^ | 14.4 ± 0.3^a^ | 15.2 ± 0.4^a^ | 15.9 ± 0.4^a^ | 14.7 ± 0.6^a^ | 15.1 ± 0.5^a^ |
| *Week 14* | 12.0 ± 0.3^b^ | 15.9 ± 0.4^acdef^ | 12.8 ± 0.5^b*^ | 11.5 ± 0.4^b*^ | 11.6 ± 0.3^b*^ | 11.2 ± 0.3^b*^ |
| FBG (mmol/L) | | | | | |  |
| *Week 10* | 4.8 ± 0.2^bcdef^ | 6.7 ± 0.2^a^ | 6.8 ± 0.4^a^ | 6.7 ± 0.3^a^ | 6.8 ± 0.3^a^ | 6.4 ± 0.1^a^ |
| *Week 14* | 4.9 ± 0.2^bf^ | 7.1 ± 0.2^acdef*^ | 4.8 ± 0.3^b*^ | 4.7 ± 0.3^b*^ | 5.0 ± 0.2^b*^ | 4.6 ± 0.3^ab*^ |
| FINS (plasma; mU/L) | | | | | | |
| *Week 10* | 36.0 ± 1.0^bcdef^ | 42.88 ± 0.3^a^ | 42.2 ± 0.7^a^ | 43.1 ± 1.3^a^ | 44.1 ± 0.3^a^ | 45.4 ± 2.3^a^ |
| *Week 14* | 38.0 ± 1.1^b^ | 46.93 ± 1.6^acdef^ | 40.2 ± 1.0^b*^ | 38.3 ± 1.0^b*^ | 38.6 ± 1.2^b*^ | 37.1 ± 0.5^b*^ |
| HOMA-IR | | | | | | |
| *Week 10* | 8.0 ± 0.2^bcdef^ | 11.8 ± 0.3^a^ | 13.6 ± 1.3^a^ | 13.2 ± 0.5^a^ | 12.1 ± 0.1^a^ | 11.5 ± 0.5^a^ |
| *Week 14* | 8.1 ± 0.4^b^ | 14.7 ± 0.4^acdef^ | 8.5 ± 0.4^b*^ | 8.1 ± 0.6^b*^ | 8.4 ± 0.2^b*^ | 7.3 ± 0.2^b*^ |
| OGTT- AUC (mmol × min) | | | | | | |
| *Week 10* | 557.3 ± 12.8^bcdef^ | 882.2 ± 79.4^a^ | 758.5 ± 35.9^a^ | 761.0 ± 18.5^a^ | 703.1 ± 13.1^a^ | 712.2 ± 13.6^a^ |
| *Week 14* | 563.0 ± 13.8^b^ | 715.8 ± 23.3^acdef^ | 600.8 ± 8.3^b*^ | 585.4 ± 46.3^b^ | 613.2 ± 18.8^b*^ | 566.6 ± 26.2^b*^ |

Abbreviations: CON, control group; EMA, combined therapies + compound-c group; EMC, combined therapies group; EXE, moderate exercise group; FBG, fasting-blood glucose; FINS, fasting insulin; HOMA-IR, homeostatic model assessment of insulin resistance; MET, metformin group; PRE, prediabetes group. Notes: Data are expressed as the mean ± standard error of the mean (*n* = 6/group). ^a^*p* < 0.05 vs. CON group; ^b^*p* < 0.05 vs. PRE group; ^c^*p* < 0.05 vs. MET group; ^d^*p* < 0.05, vs. EXE group; ^e^*p* < 0.05 vs. EMA group; ^f^*p* < 0.05 vs. EMC group; ^*^*p* < 0.05 data from week 14 vs. week 10. Two-way repeated ANOVA tests were performed to compare results before and after treatments; one-way ANOVA tests were performed to compare differences between multiple groups after treatments
